# MULTI-OMIC BIOLOGICAL AGE ESTIMATION, CORRELATION WITH WELLNESS, DISEASE PHENOTYPES: LONGITUDINAL SAMPLE OF 3558

**DOI:** 10.1093/geroni/igz038.761

**Published:** 2019-11-08

**Authors:** Nathan Price, Nathan Price, John Earls, Noa Rappaport, Laura Heath, Tomasz Wilmanski, Jennifer Lovejoy, Leroy Hood

**Affiliations:** Institute for Systems Biology, Seattle, Washington, United States

## Abstract

Biological age (BA) has been shown to be a better predictor of mortality and disease than chronological age (CA). Aging’s diverse effects are visible across many data types. We investigated BA from deep phenotyping of 3558 wellness program participants and controls. The Klemera-Doubal BA estimation algorithm was applied to genetic and longitudinal clinical laboratory, metabolomic, and proteomic assay data. BA trajectories were calculated using Generalized Estimating Equations. BA of individuals with Type-2 Diabetes averaged 6+ years greater than CA. Wellness program participation decreased individuals’ rate of aging (coefficient: -0.16, 95% CI: -0.45, 0.19), with effects dependent on sex, initial BA, and CA. Measures of metabolic health, inflammation, and toxin bioaccumulation were strong predictors across data types and sex. Deep phenotyping enabled calculation of a BA measure and a wellness program improved BA, either in absolute terms or relative to CA, showing it is modifiable.

